# Mediterranean diet adherence and synergy with acute myocardial infarction and its determinants: A multicenter case-control study in Italy

**DOI:** 10.1371/journal.pone.0193360

**Published:** 2018-03-15

**Authors:** Giuseppe La Torre, Rosella Saulle, Francesca Di Murro, Roberta Siliquini, Alberto Firenze, Massimo Maurici, Alice Mannocci, Vittoria Colamesta, Francesco Barillà, Fabio Ferrante, Luciano Agati, Collaborative group

**Affiliations:** 1 Department of Public Health and Infectious Diseases, Sapienza University of Rome, Rome, Italy; 2 Department of Public Health Sciences and Pediatrics Torino University, Turin, Italy; 3 Department of Sciences for Health Promotion and Mother-Child Care "G. D'Alessandro", University of Palermo, Palermo, Italy; 4 Department of Biomedicine and Prevention, University of Rome Tor Vergata, Rome, Italy; 5 Department of Cardiovascular, Respiratory, Nephrological, Anesthesiological and Geriatric Sciences, Sapienza University of Rome, Rome, Italy; 6 Department of Cardiology, Policlinico Umberto I, Sapienza University of Rome, Rome, Italy; Azienda Ospedaliero Universitaria Careggi, ITALY

## Abstract

**Background:**

Cardiovascular diseases are the leading causes of mortality and morbidity in Western countries. The possible synergistic effect of poor adherence to a Mediterranean diet (MD) and other risk factors for acute myocardial infarction (AMI) such as hypertension, cholesterol, ever smoker, BMI> 25, diabetes, has not been deeply studied.

**Design:**

Case-control study.

**Methods:**

Patients with first AMI and controls from four tertiary referral Italian centers were screened for enrolment. Dietary information was collected through a questionnaire and a MD adherence score was calculated. Physical activity and smoking habits were also registered. The Synergy Index was calculated according to Rothman.

**Results:**

127 cases and 173 controls were enrolled. The analysis was conducted using a dichotomous variable for the MD score with values ≥7 representing good adherence. Multivariate analysis showed the following variables associated to AMI: ever smoker (OR = 2.08), diabetes (OR = 1.42), hypertension (OR = 2.08), hypercholesterolemia (OR = 2.47), BMI> 25 (OR = 1.99), while a protective effect emerged both in subjects scoring > 7 on the MD score (OR = 0.55) and in subjects resident of Southern Italy (OR = 0.38). A synergistic effect does exist between poor adherence to the MD and the following risk factors: hypertension, hypercholesterolemia, BMI >25, diabetes and being a resident in central and northern Italy.

**Conclusion:**

Synergy between heart disease risk factors and MD underlines the need to enlarge the list of known modifiable cardiovascular risk factors to include and promote adherence to Mediterranean dietary habits.

## Introduction

Myocardial infarction (MI) is caused by acute ischemia that persists long enough to cause cell necrosis. It is, therefore, characterized by irreversible anatomical alterations of the myocardium. In most trans-mural infarcts, early coronary angiography, performed in the immediate hours post MI occurrence, has allowed us to document complete coronary occlusion. In most cases of Acute Myocardial Infarction (AMI) this is due to a thrombosis, and, with much lower frequency, can be caused by coronary spasm. Both events occur almost exclusively, at the level of atherosclerotic lesions [1]. Cardiovascular diseases are the leading causes of mortality and morbidity in Western countries [2]. However, some epidemiological studies [3–5] report relatively high homogeneity to the incidence rate of cardiovascular diseases in relation to different geographical areas. A low incidence of coronary heart disease was found in southern Europe and in particular in countries bordering the Mediterranean, such as France, Spain, Greece and Italy, compared to northern Europe and the United States. The geographical variability was attributed to environmental factors, different lifestyles and in particular to different eating habits, with Mediterranean countries traditionally reflecting the dietary habits akin to the "Mediterranean diet". Adherence to the Mediterranean Diet (MD) seems to provide significant protection against death from any cause [6–8] and the incidence of major chronic degenerative diseases (reduction of cardiovascular events [9, 10] and cancer incidence). However, there is sufficient evidence that even in Norther countries, high quality diets in line with current recommendations may reduce the risk of CV events [11].

High adherence to a Mediterranean diet profile, is associated with a reduced hazard from perceived stress; this psychological stress is greater in women compared to men. The perception of illness is important in the context of perceived stress associable to atrial fibrillation (AF). High adherence to the Mediterranean diet can be warranted in order to achieve a lower index of psychological distress in AF patients [12]. According to the LYON study, Mediterranean diet decreases the death rate from coronary artery disease (CAD) by 50% [13].

The relationship between dietary factors and coronary heart disease (CHD) has been a major goal of health research since the early 1960s. Keys and Aravanis [14] connected the Mediterranean diet to lower CHD incidence, by observing that the traditional food model among southern European populations was based on the Mediterranean diet. The typical Mediterranean diet is characterized by a high consumption of olive oil, fruits, vegetables, whole grains and cereals, legumes, fish, nuts, poly and monounsaturated fats- In particular, the oleic acid content in olive oil and the limited intake of "trans" fats, saturated fat and cholesterol [15] through a lower consumption of red meat, dairy products and sweets as well as a moderate intake of red wine during meals completes the dietary profile. The Mediterranean diet is considered a dietary model to pursue—both in primary as well as secondary prevention [8, 16]—because it is able to modify the cardiovascular risk profile of patients [17–19] guiding them towards the achievement and maintenance of a good state of health and even promote longevity [16. 20].

Smoking and passive smoking have been identified as modifiable risk factors for acute myocardial infarction (AMI) and acute coronary syndrome (ACS). The INTERHEART study [21] is a case-control investigation on AMI conducted in 52 countries (Africa, Asia, Australia, Middle East and North and South America). This study showed that tobacco use is one of the most important causes of global AMI, especially in men. Smokers have an increased risk of nonfatal myocardial infarction (OR 2,95, IC 95%: 2,77–3,14, p <0,0001) compared to non-smokers; their risk is increased by 5.6% for each cigarette smoked. The OR associated with a former smoker, drops to 1.87 (95% CI: 1,55–2,24) within 3 years after they have stopped smoking. A residual risk remains even more than 20 years after quitting (1.22, 1.09–1.37). In a large multiethnic population [22] the odds ratio (OR) for AMI in smokers was 2.95 compared to non-smokers. Tobacco use is the leading preventable cause of death worldwide, responsible for 5 million deaths each year or 12% of all deaths. In addition, 29% of coronary vascular disease deaths are attributable to tobacco [23].

While it is well recognized that there is a synergy among the nutrient-rich foods included in the MD that has a positive impact in the changes in intermediate pathways of cardiometabolic risk (i.e., blood lipids, insulin sensitivity, resistance to oxidation, inflammation, and vasoreactivity) [24], little is known on the synergism between MD and the classical risk factors for AMI. Pitsavos et al. [25], working on hypercholesterolemic patients, observed a synergistic effect of the combination of the MD with statin treatment on coronary risk. They found that following the MD pattern was associated with a reduction of the risk of developing acute coronary syndromes in diabetic, physically inactive, and obese subjects.

Moreover, in describing cardiodiabesity, i.e. the relationship between type 2 Diabetes Mellitus (T2DM), obesity, the metabolic syndrome (MetS) and cardiovascular disease (CVD), Garcia-Fernandez et al [26], stated that specifically designed studies should be carried out to better evaluate these outcomes simultaneously with the aim of determining the synergy of interventions in several health parameters involved in cardiodiabesity.

The objective of this study was to carry out a case-control study that gauges the association between Mediterranean diet adherence, smoking habits and nonfatal myocardial infarction. Furthermore, it also attempts to assess the existence of a synergistic effect between the different risk factors for AMI, by combining poor adherence to the Mediterranean diet (score <7) with the following risk factors for AMI: hypertension, cholesterol, ever smoker, BMI> 25, diabetes and to being an inhabitant of central and northern Italy.

## Materials and methods

The association between Mediterranean diet, tobacco smoke and AMI was evaluated through a multicenter case-control study coordinated by the Department of Public Health and Infectious Diseases at Sapienza University of Rome. Patients with first AMI and controls from four tertiary referral centers "Sapienza" University Hospital, Rome, "Tor Vergata”, University Hospital, Roma (Central Italy), “San Giovanni Battista” Hospital, Turin (Northern Italy) and “Policlinico Giaccone”, Palermo (Southern Italy)] were screened for study enrolment. Patients were included if they were older than 18 years, met ECG criteria for AMI and were successfully treated by primary coronary intervention within 12 h of symptom onset (i.e. chest pain). Exclusion criteria included pulmonary oedema and/or cardiogenic shock persisting after the first week of hospitalization, prior coronary revascularization or myocardial infarction, The study was approved by the Ethics Committee of the Policlinico Umberto I Hospital, in line with the Declaration of Helsinki protocol (1989). The diagnosis of AMI referred to the Guidelines of the Italian Journal of Cardiology Guide [27].

### Identification of controls

Controls were selected from patients without AMI, in the departments of Orthopaedics and Ophthalmology of the same hospitals and matched to cases by sex and age (case-control study with hospital controls). Controls were patients from the same area, that were admitted to the same hospitals in the same period of the cases for a wide spectrum of acute non-neoplastic conditions, not related to known AMI risk factors or dietary modification. Controls with previous major cardiovascular events were not included in the study. Among controls, 45% had eye disorders, 25% had traumas, 30% had non-traumatic orthopaedic disorders.

All the patients gave written consent.

Normal eating habits of participants were assessed using the validated Food Frequency Questionnaire, (FFQ), which included 12 items corresponding to the12 elemental characteristics of the Mediterranean diet: carbohydrates, vegetables, fruit, milk, extra virgin olive oil, white meat, red meat, sausages, fish, eggs, legumes and sweets [28]. The consumption frequency for a particular food item was reported on a nine-level scale (never or almost never, 1–3 times a month, once a week, 2–4 times a week, 5–6 times a week, once a day, 2–3 times a day, 4–6 times a day, and more than six times per day). Participants received a score of 1 in the event that the consumer fitted the reference frequencies and a score of 0 if not. For each participant, the 0–12 score was constructed by adding the scores obtained for 12 groups of foods. Adherence to the Mediterranean diet was assessed by a score created by Trichopoulou et al [16]. According to daily intake, as score for each participant was calculated upon consuming a given food within the Mediterranean diet model, on a daily, weekly or monthly basis. To each of the 12 components was assigned a score ranging from 0 to 1 according to the reference daily / weekly frequency of the Pyramid of the Modern Mediterranean Diet, such as: *Main meals*: 1–2 servings of fruit a day (1 item); > = 2 vegetable / vegetable servings (2nd item); 1–2 servings of bread, pasta, rice, couscous and other grains (3rd item). *Daily consumption*: 23 servings of milk(4th item);3–4 servings of olive oil (5th item). *Weekly consumption*: 1–2 servings of poultry (6th item); > = 2 fish (7th item); 2–4 portions of eggs (8th item); > = servings of legumes (9th item). *More coverage weekly*:< = portion of sausages (10th item); < = 2 servings of red meat (11th item); < = 2 portions of sweets (12° item). The total score of the Mediterranean diet ranged from 0 (minimum adherence to the traditional Mediterranean diet) to 9 (maximum adhesion). People who consumed beneficial components (vegetables, legumes, fruits, cereals, fish) below the average consumption (score of the Mediterranean Diet <7), had been assigned a value of 0, and a value of 1 if they consumed these beneficial components above the average consumption (score of the Mediterranean Diet> 7). Adherence to the Mediterranean diet was ranked from a low adherence (score <7) to a high adherence (score ≥7).

For smoking habits, patients were divided into three categories: current smoker, exsmoker and never smoker. A new category, the *ever smoker*, was also created, this included both current smokers and ex-smokers together. Ever smokers were asked at what age they started smoking, if they smoked every day and the amount of cigarettes they smoked in a day. Ex-smokers were asked at what age they stopped smoking. Exposure to secondhand smoke at home and / or at work, was also investigated asking whether they had ever been exposed to secondhand smoke by one or more people, for how many years and for how many hours per day. In particular, ever smokers were subjected to testing on tobacco dependence through the Fagerström test [29] and their motivation to quit smoking assessed through the Mondor test [12]. All participants (cases and controls) completed the SF-12 questionnaire for the evaluation of quality of life [30]. The SF-12 questionnaire is characterized by brevity (the average person takes no more than 10 minutes to complete) and precision (the instrument is valid and reproducible). The SF-12 consists of 12 items that produce two measures related to two different aspects of health: physical and mental health. It has four scales (physical functioning, role physical, role emotional state, mental health) each measured by 2 items and another 4 scales, each measured by one item (bodily pain, vitality, social activities and health in general). The subject is asked to respond on how they feel and how they manage to carry out usual activities on the day they fill out the questionnaire and during the previous four weeks. In addition, data on physical activity (using the Italian version of the International Physical activity Questionnaire) [31] and other relevant family health conditions were collected: diabetes, hypertension, hypercholesterolemia, hypertriglyceridemia, stroke, cardiovascular diseases, in particular myocardial infarction and obesity. Patients were asked their weight and height, and their BMI was calculated. All patients signed an informed consent form before compiling.

### Statistical analysis

For assessing the sample size, EpiCalc2000 was used. The following parameters were considered:

Ratio of cases to controls: 1

OR to detect: 0.50

Proportion (%) controls exposed: 60%

Significance: 0.05

Power: 80%

On the basis of these calculations we needed to recruit 132 cases and 132 controls.

Absolute and relative frequency distribution as well as contingency tables were created. The differences between cases and controls were analyzed using parametric and nonparametric tests. To describe the cut off score of MD and to evaluate the best AMI predictive score, a ROC curve (Receiver Operating Characteristic) was used. The differences between groups (cases and controls) were evaluated using nonparametric tests such as Chi-square and Mann-Whitney. Subsequently, a multivariate analysis was conducted, using a logistic regression model to analyze which risk factors were independently associated with AMI. A stepwise procedure with backward elimination was used. The variables presented in step 1 were those that had a p level of <0:20 in the univariate analysis. The results are presented as ORs and confidence intervals (CI) set at 95%. With regard to the patients’ place of residence, the sample was divided into central, northern and southern Italy residents (Rome, Turin and Palermo). Finally, to assess the presence of any synergism between the MD score and various risk factors, the ORs were calculated each time a combination between a singular risk factor and Mediterranean Diet score of <7 was encountered. A synergistic effect was created by combining a risk factor to the MD score. When hypertension was combined with MD score, a MD ≥7 score was given the value of 0 and <7 was given a value of 1, equivalent to a hazardous condition.

**Fig 1:**
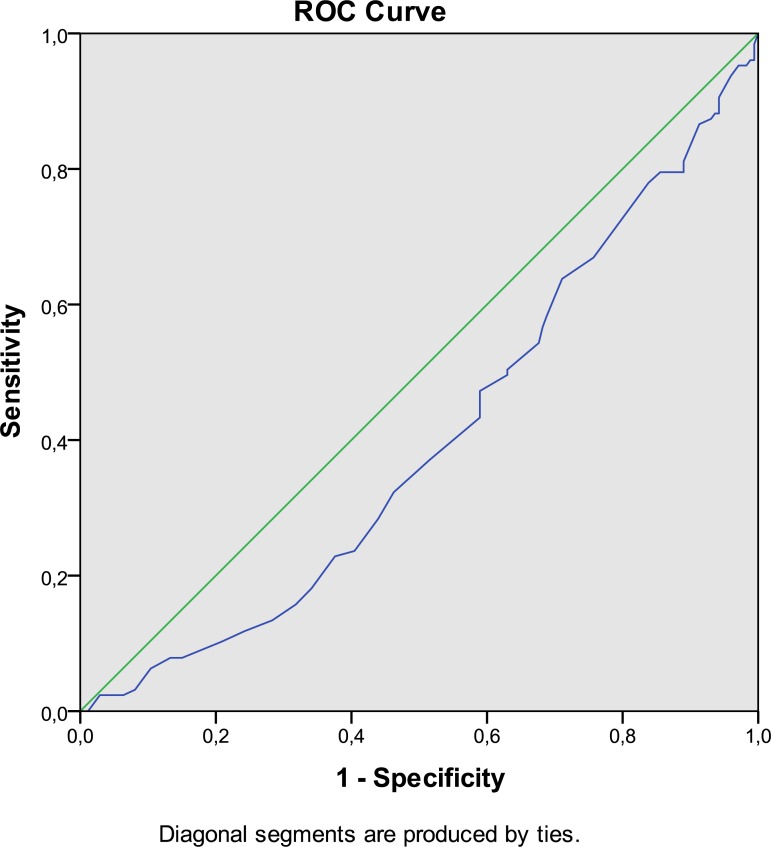
The receiver operating characteristic curve of the Mediterranean diet score.

For hypertension a value of 10 (equivalent to 1) was signed in the event of its presence. The statistical analysis was performed using the SPSS package for Windows, version 23.0. The level of significance was set at p <0.05. From the analysis of the ROC curve (Fig 1), it can be seen that the MD score has a protective effect against AMI (blue curve below the bisector of the angle), with AUC = 0.401 (p = 0.003). The MD score value that best predicts the outcome was equal to 6.75 (sensitivity = 57%, specificity = 59%) (Fig 1).

A dichotomous variable for a value greater than or equal to 7 (good adhesion to MD) and under 7 (poor adherence to MD) was considered. The synergism index was calculated to assess whether there was a synergistic effect between the MD score and all other risk factors for AMI such as hypertension, cholesterol, smoking (considering the category ever smoker), BMI> 25, diabetes, and being a resident of central and northern Italy. The Sicily Region (equivalent to South Italy) was given a value of 0 because it was a protective factor for AMI. Using these variables in a logistic regression model the OR values were obtained and an index of synergy then calculated. For this calculation, and according to Rothman, the following formula was used: S = (OR11–1) / [(OR01 OR10 +) - 2] [32], where OR11 is the joint effect of two risk factors and OR10 and OR01 are equal to a risk factor in the absence of the other. A value of S equal to unity was interpreted as indicating additivity, while a higher unit value is indicative of synergism. Other measures of additive interactions were calculated: relative excess risk due to interaction defined as RERIOR = OR11—OR10—OR01 + 1; proportion of risk in doubly exposed groups attributable to interaction defined as

$$AP=\frac{RR_{11}-RR_{10}-RR_{01}+1}{RR_{11}}$$ (1)

proportion of the joint effects of both exposures attributable to interaction defined as

$$AP^{*}=\frac{RR_{11}-RR_{10}-RR_{01}+1}{RR_{11}-1}$$ (2)

## Results

One hundred and twenty seven cases and 173 controls participated in the study. Table 1 shows the socio-demographic characteristics of the sample.

**Table 1:** Characteristics of cases and controls.

Variable	Controls N(%)	Cases N(%)	p-value
*Gender*			
Male	129 (74.6)	97 (76.4)	0.719*
Female	44 (25.4)	30 (23.6)	
Age (years, median) (SD)	59.6 (11)	60.3 (10.4)	0.978^
*Full time job*			
No	115(66)	73(57)	0.110*
Yes	58(33)	54(42)	
*Married-cohabitant*			
No	45(26)	31(24)	0.750*
Yes	128(74)	96(76)	
*Cohabiting person*			
0–2	91 (52.9)	57 (45.2)	0.191*
> = 3	81 (47.1)	69 (54.8)	
*Children*			
0–1	49(29)	31(24.8)	0.424*
> = 2	120(71)	94(75)	
*Qualification*			
Middle-elementary schools	93(54)	73(57)	0.520*
Degree/ high school	80(46)	54(42)	
*Region*			
North-Central	52(30)	67(53)	< 0.0001*
South	121(70)	60(47)	
*PCS average (SD)*	45(11)	43(12)	0,208^
*MCS average (SD)*	44(10)	45(10)	0,351^

Chi-square

^ Mann-Whitney test

PCS = Physical component summary MCS = Mental Component Summary

There were no significant differences between cases and controls with respect to gender, age, occupation, marital status, number of cohabiting persons and children, and educational qualification. Regression analysis shows that living in Sicily, was a protective factor for AMI.

There is a significant difference between cases and controls (Table 2) regarding risk factors such as hypertension, hypercholesterolemia, BMI> 25, MD score> = 7, and tobacco smoking (ever smoker). The Multivariate analysis (Table 3), conducted using a multiple logistic regression model, highlights the association between AMI and the following variables: ever smoker, diabetes, hypertension, hypercholesterolemia and BMI> 25. Southern Regions and a MD≥ 7 score are protective against AMI.

**Table 2:** Differences in risk factors between cases and controls.

Variable	Controls N(%)	Cases N(%)	p-value
*Ever smoker*			
No	64(37)	28(22)	0,006*
Yes	109(63)	99(78)	
*Diabetes*			
No	133(77)	89(70)	0,185*
Yes	40(23)	38(30)	
*Hypertension*			
No	98(57)	53(41)	0,011*
Yes	75(43)	74(58)	
*Hypercholesterolemia*			
No	126(73)	66(52)	<0,001*
Yes	47(27)	61(48)	
*BMI >25 Kg/m*^*2*^			
No	66(38)	30(24)	0,008*
Yes	107(62)	97(76)	
*MD score > = 7*			
No	87(50)	82(65)	0,014*
Yes	86(50)	45(35)	
*Physical activity*			
No	135(78)	92(72)	0,26*
Yes	38(22)	35(28)	

Chi-square

**Table 3:** Results of multivariate analysis (outcome variable = case of AMI).

Variable	ORcrude (95% CI OR)	ORadj (95% CI OR)
*Ever Smoker*		
No	1	1
Yes	2.08 (1.23–3.49)	2.15 (1.21–3.80)
*Diabetes*		
No	1	1
Yes	1.42 (0.84–2.38)	1.75 (0.95–3.25)
*Hypertension*		
No	1	1
Yes	1.82 (1.15–2.90)	1.85 (1.05–3.23)
*Hypercholesterolemia*		
No	1	1
Yes	2.47 (1.53–4.01)	2.31 (1.36–3.90)
*BMI >25 Kg/m*^*2*^		
No	1	1
Yes	1.99 (1.19–3.32)	1.99 (1.12–3.57)
*MD score ≥7*		
No	1	1
Yes	0.55 (0.34–0.88)	0.56 (0.32–0.97)
*Region*		
North-Central	1	1
South	0.38 (0.24–0.62)	0.31 (0.18–0.55)

Table 4 shows the ORs calculated with the logistic regression analysis to evaluate the SI.

**Table 4:** OR calculation for obtaining the index of synergism.

	Hypertension	Hypercholesterolemia	Ever smoker	BMI over 25 *Kg/m*^*2*^	Diabetes	Centralnorth
OR_01_	1,4	2,3	0,77	1,72	1,37	0,68
OR_10_	1,37	3,34	1,07	1,93	0,89	1,75
OR_11_	3,77	5,11	2,6	3,88	5,74	2,24

Concerning the Index of synergy and other measures of additive interaction (Table 5), a particularly excessive risk is represented in cases of high interaction between diabetes and MD score <7 (RERIOR = 4.48), and between hypertension and MD score <7 (RERIOR = 2). There is always interaction between score MD <7 and the other risk factors for AMI. The risk of double proportion attributable to the interaction of exposure (Attributable Proportion, AP) is 78% for the interaction between diabetes and MD score <7, 53% for the interaction between hypertension and MD score< 7, 36.2% if interaction is between Centre-North and MD score <7, and 31.7% taking into account BMI and MD score <7. The combined effect of the proportion attributable to a double exposure interaction (AP) is 94.5% if we consider the interaction between diabetes and MD score <7%, 72% for the interaction between hypertension and MD score <7%, and 65.3% for the interaction between being a resident of the Center-North and MD score <7.

**Table 5:** Index of synergy and other measures of additive interaction.

Variables		Index of synergism	RERI_OR_	AP	AP+
Hypertension	Score DM< 7	3.59	2	53%	72.2%
Hypercholesterolemia	Score DM< 7	1.12	0.47	9.2%	11.4%
Ever_smoker	Score DM< 7	-10	1.76	n.a.	n.a.
BMI> 25	Score DM< 7	1.74	1.23	31.7%	42.7%
Diabetes	Score DM< 7	18.23	4.48	78%	94.5%
North-Central	Score DM< 7	2.88	0.81	36.2%	65.3%

## Discussion

This work represents the first multicenter case-control study in Italy on the association between adherence to MD and AMI. Statistically significant differences were found between cases and controls with respect to risk factors such as diabetes, hypertension, hypercholesterolemia, low adherence to the Mediterranean diet (score <7), BMI> 25, tobacco smoke (ever smoker) and being a resident in one of the central and northern Italian regions. Moreover, the results show that there is a synergistic effect between MD score and many risk factors for AMI, such as hypertension, hypercholesterolemia, BMI> 25, diabetes, as well as living in a region of the Centre-North. Comparing the present study with another study, we considered a national case-control study which also investigated the association between the Mediterranean diet and AMI [33]; the latter is not a multicenter study and does not investigate other risk factors for AMI outside of the MD score, so we cannot evaluate the synergistic effect of multiple risk factors for AMI. Another case-control study investigating the metabolic syndrome, adherence to Mediterranean diet and 10-year cardiovascular disease incidence (The ATTICA study) [34] identified five factors associated with a higher risk of AMI, namely increased weight, hypertension, dyslipidemia, hyperglycemia, and increased inflammatory markers. However, the synergistic effect that exists between them was not evaluated. Finally the INTERHEART study [21] only takes tobacco smoke into account, and thus doesn’t consider the synergistic effect between several modifiable risk factors.

Our study is in agreement with the results of a recent systematic review carried out by Rosato et al [35] in which they found a protective effect of the MD on AMI (pooled RR for CHD/AMI risk = 0.70; 95% CI 0.62–0.80), based on 11 studies.

After the comparison with other national and international studies, one can say that this study has the advantage of being a multi-center study, and had the opportunity to cover different Italian Regions with different adherence to the MD pattern. It is the first in Italy, with cases and hospital controls enrolled in northern, central and southern Italian regions thus allowing for a territorial comparison also. Unlike other studies, it evaluates the synergistic effect between a low MD (<7) score and the various risk factors for AMI (high blood pressure, cholesterol, ever smoker, BMI> 25, diabetes, and being an inhabitant of central and northern Italy, and suggests that this synergism index is super additive if multiple factors co-exist with low adherence to MD.

This study has important strengths such as the rigorous and validated methodology used, as well as the existence of previously published validation studies, the study design and the multicenter characteristics. One limitation of the study, such as all the case-control studies, is due to the recall bias, because most of the questionnaire were based on the self-reporting. Another limitation is due to the possibility to investigate exclusively on the traditional risk factors (derived from the Framingham study such as hypertension, diabetes, and smoking) and to the impossibility (especially on controls group) to inquired on the non-traditional ones, linked to the CKD itself or to the hyperuricemia, where elevated serum uric acid levels are strongly associated with cardiovascular risk [36–39]. Moreover, in our study we are not able to exclude that a generally healthier lifestyle might play a role in the inverse association of adherence to the MD with AMI risk. So, we paid a particular attention to adjust for the potential confounding of covariates associated with AMI risk, such as BMI, smoking, diabetes, hypercholesterolemia, hypertension and geographical localization.

Unfortunately, and ironically, in modern times there is little or no adherence to the Mediterranean diet in the land where it was discovered, as described in a cross sectional survey among a younger generation of adolescents in the heart of the Cilento region in Southern Italy [40]. The study investigated dietary habits among young people in rural Mediterranean areas, exactly where the health benefits of the Mediterranean diet were discovered by Ancel Keys, and were traditionally culturally adhered to. In that study, adherence to the MD was appraised according to a 0–10 scale, taking into account also another study, conducted in the Mediterranean area (Spain), carried out by Martinez-Gonzalez at al [41], in which the authors used a 9-point score to appraise adherence to the MD.

Remarkably 63.8% had a score of under six, indicating that the majority of the students did not particularly follow a Mediterranean diet whilst only 36.2% (n. 371) exceeded a score of six, adhering to it in varying degrees.

Accordingly, we need protocols for overweight/obese patients, as shown by Pancallo et al., who highlighted the efficacy of treatment protocols in overweight/obese patients [42]. The authors extracted data from 762 medical records of patients with a BMI≥25 (2009–2012). BMI average on beginning of treatment was 30.26 and by the end of treatment it had dropped to 28.37. Just by adopting dietary changes 55.2% of the sample had lost up to 4.9 kg.

The primary prevention of AMI could be mainly implemented using a double target: a) the adult population; b) the children population. As far as concerns the first population, there is a strong evidence that the prevention with a specific focus on diet is effective in reducing the incidence of AMI. Stamler and coll., almost 50 years ago started the Coronary Prevention Evaluation Programme with the aim of assessing combined nutritional-hygienic means for the correction of five coronary risk factors, such as cigarette smoking, hypercholesterolaemia, hypertension, obesity and physical inactivity. Their approach was based on the hypotesis that the coronary heart disease epidemic is mainly associated with faulty living habits that act synergistically to intensify risk [43].

On the other hand, school-based interventions targeted on the childhood population for preventing obesity and promoting physical activity are well recognized as effective [44–46].

In conclusion, synergy between heart disease risk factors and MD underlines the need to enlarge the list of known modifiable cardiovascular risk factors to include and promote adherence to Mediterranean dietary habits.

## References

[1] Harrison TR. The cardiovascular system diseases Principles of Internal Medicine. Vol. 2, 16th ed., McGraw Hill, 2005 ISBN 88-386-2999-4.

[2] Mozaffarian D, Benjamin EJ, Go AS, Arnett DK, Blaha MJ, Cushman M et al Heart disease and stroke statistics-2015 update: a report from the American Heart Association. Circulation 2015;131:e29–e322 doi: 10.1161/CIR.0000000000000152 25520374 10.1161/CIR.0000000000000152

[3] Keys A, Menotti A, Karoven MI. The diet and the 15-year death rate in the Seven Countries Study. Am J Epidemiol 1986 124:903–15. 3776973 10.1093/oxfordjournals.aje.a114480

[4] Tunstall-Pedoe H, Kuulasmaa K, Mähönen M, Tolonen H, Ruokokoski E, Amouyel P. Contribution of trends in survival and coronary-event rates to changes in coronary heart disease mortality: 10-year results from 37 WHO MONICA project populations. Monitoring trends and determinants in cardiovascular disease. Lancet 1999; 353(9164):1547–57 10334252 10.1016/s0140-6736(99)04021-0

[5] Masiá R, Pena A, Marrugat J, Sala J, Vila J, Pavesi M, et al High prevalence of cardiovascular risk factors in Gerona, Spain, a province with low myocardial infarction incidence. REGICOR investigators. J Epidemiol Comm Health 1998;52:707–15. 10.1136/jech.52.11.707 PMC1756647 10396503

[6] Sacks FM, Katan M. Randomized clinical trials on effects of dietary fat and carbohydrate on plasma lipoproteins and cardiovascular disease. Am J Med. 2002; 113 (Suppl 9B):13S–24S 12566134 10.1016/s0002-9343(01)00987-1

[7] Tande DL, Hotchkiss L, Cotugna N. The association between blood lipids and the food guide pyramid: findings from the Third National Health and nutrition examination survey. Prev Med 2004; 38 452–7 doi: 10.1016/j.ypmed.2003.11.018 15020178 10.1016/j.ypmed.2003.11.018

[8] Ricco A, Chiaradia G, Piscitelli M, La Torre G. The effects of Mediterranean Diet on Cardiovascular diseases: a systematic review. Ital J Public Health 2007; 5 (4) 2:119–27.

[9] Liu S, Stampfer MJ, Hu FB, Giovannucci E, Rimm E, Manson JE et al Whole-grain consumption and risk of coronary heart disease: results from the Nurses’ Health Study. Am J Clin Nutr. 1999; 70 (3):412–9. 10479204 10.1093/ajcn/70.3.412

[10] Fung TT, Willett WC, Stampfer MJ, Manson JE, Hu FB. Dietary patterns and risk of coronary heart disease in women. Arch Intern Med 2001; 161(15):1857–62. 11493127 10.1001/archinte.161.15.1857

[11] Hlebowicz J, Drake I, Gullberg B, Sonestedt E, Wallström P, Persson M et al A high diet quality is associated with lower incidence of cardiovascular events in the Malmö diet and cancer cohort. PLoS One. 2013 8 5;8(8):e71095 doi: 10.1371/journal.pone.0071095 23940694 10.1371/journal.pone.0071095 PMC3733649

[12] La Torre G (Editor). Smoking Prevention and Cessation. Springer Science+ Business Media, New York 2013

[13] de Lorgeril M, Salen P, Martin JL, Monjaud I, Delaye J, Mamelle N. Mediterranean diet, traditional risk factors, and the rate of cardiovascular complications after myocardial infarction: final report of the Lyon Diet Heart Study. Circulation. 1999 2 16;99(6):779–85. 9989963 10.1161/01.cir.99.6.779

[14] Keys A, Aravanis C, Blackburn H et al (eds). A multivariate analysis of death and coronary heart disease Harvard University Press Cambridge Mass, 1980; pp. 1–381.

[15] Martinez-Gonzalez MA and Sanchez-Villegas A. The emerging role of Mediterranean diets in cardiovascular epidemiology: monounsaturated fats, olive oil, red wine or the whole pattern? Eur J Epidemiol 2004;19(1):9–13. 15012018 10.1023/b:ejep.0000013351.60227.7b

[16] Trichopoulou A, Kouris-Blazos A, Wahlqvist ML, Gnardellis C, Lagiou P, Polychronopoulos E et al Diet and overall survival in elderly people. BMJ 1995;311:1457–60 8520331 10.1136/bmj.311.7018.1457 PMC2543726

[17] Ryan M, McInerney D, Owens D, Collins P, Johnson A, Tomkin GH. Diabetes and the Mediterranean diet: a beneficial effect of oleic acid on insulin sensitivity, adipocyte glucose transport and endothelium-dependent vasoreactivity. Q J med 2000;93(2):85–91. 10.1093/qjmed/93.2.85 10700478

[18] Ambring A, Friberg P, Axelsen M, Laffrenzen M, Taskinen MR, Basu S, et al Effects of Mediterranean inspired diet on blood lipids, vascular function and oxidative stress in healthy subjects. Clin Sci 2004;106(5):519–25 doi: 10.1042/CS20030315 14683522 10.1042/CS20030315

[19] Ramallal R, Toledo E, Martínez-González MA, Hernández-Hernández A, García-Arellano A, Shivappa N et al Dietary Inflammatory Index and Incidence of Cardiovascular Disease in the SUN Cohort. PLoS One. 2015 9 4;10(9):e0135221 doi: 10.1371/journal.pone.0135221 26340022 10.1371/journal.pone.0135221 PMC4560420

[20] World Health Organization Study Group. Diet, nutrition, and the prevention of chronic diseases. Geneva: WHO, 2003, Tech rep Ser 2003;916 12768890

[21] Yusuf S, Hawken S, Ounpuu S, Dans T, Avezum A, Lanas F et al Effect of potentially modifiable risk factors associated with myocardial infarction in 52 countries (the INTERHEART study): case-control study. Lancet 2004;364: 937–52 doi: 10.1016/S0140-6736(04)17018-9 15364185 10.1016/S0140-6736(04)17018-9

[22] La Torre G, Saulle R, Nicolotti N, de Waure C, Gualano MR, Boccia S. From nicotine dependence to genetic determinants of smoking In Smoking Prevention and Cessation, La Torre G, Ed., pp. 1–21, Springer, London, UK, 2013

[23] WHO Global Report: Mortality Attributable to Tobacco. http://whqlibdoc.who.int/publications/2012/9789241564434_eng.pdf (5 September 2013).World Health Organisation, 2012

[24] Jacobs DR Jr, Gross MD, Tapsell LC. Food synergy: an operational concept for understanding nutrition. Am J Clin Nutr 2009;89:1543S–1548S doi: 10.3945/ajcn.2009.26736B 19279083 10.3945/ajcn.2009.26736B PMC2731586

[25] Pitsavos C, Panagiotakos DB, Chrysohoou C, Skoumas J, Papaioannou I, Stefanadis C et al The effect of Mediterranean diet on the risk of the development of acute coronary syndromes in hyperchotesterolemic people: a case-control study (CARDIO2000). Coron Artery Dis. 2002;13:295–300. 12394655 10.1097/00019501-200208000-00008

[26] García-Fernández E, Rico-Cabanas L, Rosgaard N, Estruch R, Bach-Faig A. Mediterranean Diet and Cardiodiabesity: A Review. Nutrients. 2014; 6(9): 3474–3500 doi: 10.3390/nu6093474 25192027 10.3390/nu6093474 PMC4179172

[27] Thygesen K, Alpert JS, White HD. Guidelines Universal definition of myocardial infarction. G Ital Cardiol 2008; 9 (3): 209–24

[28] Martin-Moreno JM1, Boyle P, Gorgojo L, Maisonneuve P, Fernandez-Rodriguez JC, Salvini S et al Development and validation of a food frequency questionnaire in Spain. Int J Epidemiol 1993;22:512–9 8359969 10.1093/ije/22.3.512

[29] Heatherton TF, Kozlowski LT, Frecker RC, Fagerström KO. The Fagerström test for nicotine dependence: a revision of the Fagerstrom Tolerance Questionnaire. Br J addict 1991; 86(9): 1119–27. 1932883 10.1111/j.1360-0443.1991.tb01879.x

[30] Ware J Jr, Kosinski M, Keller SD. A 12-Item Short-Form Health Survey: construction of scales and preliminary tests of reliability and validity. Med Care. 1996 3;34(3):220–33. 8628042 10.1097/00005650-199603000-00003

[31] Mannocci A, Di Thiene D, Del Cimmuto A, Masala D, Boccia A, De Vito E et al International Physical Activity Questionnaire: validation and assessment in an Italian sample. Ital J Public Health 2010; 8(7): 369–376.

[32] Rothman KJ. The estimation of synergy or antagonism. Am J epidemiol 1976;103(5):506–11. 1274952 10.1093/oxfordjournals.aje.a112252

[33] Turati F, Pelucchi C, Galeone C, Praud D, Tavani A, La Vecchia C. Mediterranean diet and non-fatal acute myocardial infarction: a case–control study from Italy. Public Health Nutr. 2015 3;18(4):713–20. doi: 10.1017/S1368980014000858 25327630 10.1017/S1368980014000858 PMC10273000

[34] Kastorini CM, Panagiotakos DB, Georgousopoulou EN, Laskaris A, Skourlis N, Zana A et al Metabolic syndrome and 10-year cardiovascular disease incidence: The ATTICA study. Nutr Metab Cardiovasc Dis 2016; 26(3):223–31. doi: 10.1016/j.numecd.2015.12.010 26803591 10.1016/j.numecd.2015.12.010

[35] Rosato V, Temple NJ, La Vecchia C, Castellan G, Tavani A, Guercio V. Mediterranean diet and cardiovascular disease: a systematic review and meta-analysis of observational studies. Eur J Nutr. 2017 11 25. 10.1007/s00394-017-1582-0 29177567

[36] Edwards NL. The role of hyperuricemia in vascular disorders. Current opinion in rheumatology. 2009;21:132–137. doi: 10.1097/BOR.0b013e3283257b96 19339923 10.1097/BOR.0b013e3283257b96

[37] Kleber ME, Delgado G, Grammer TB, Silbernagel G, Huang J, Krämer BK et al Uric Acid and Cardiovascular Events: A Mendelian Randomization Study. J Am Soc Nephrol 2015;26:2831–2838. doi: 10.1681/ASN.2014070660 25788527 10.1681/ASN.2014070660 PMC4625666

[38] Feig DI, Kang DH, Johnson RJ. Uric acid and cardiovascular risk. The New England journal of medicine. 2008;359:1811–1821. doi: 10.1056/NEJMra0800885 18946066 10.1056/NEJMra0800885 PMC2684330

[39] Gagliardi AC, Miname MH, Santos RD. Uric acid: A marker of increased cardiovascular risk. Atherosclerosis. 2009;202:11–17. doi: 10.1016/j.atherosclerosis.2008.05.022 18585721 10.1016/j.atherosclerosis.2008.05.022

[40] Saulle R, Del Prete G, Stelmach-Mardas M, De Giusti M, La Torre G. A breaking down of the Mediterranean diet in the land where it was discovered. A cross sectional survey among the young generation of adolescents in the heart of Cilento, Southern Italy. Ann Ig 2016;28(5):349–59. 10.7416/ai.2016.2115 27627666

[41] Martínez-González MA, García-López M, Bes-Rastrollo M, Toledo E, Martínez-Lapiscina EH, Delgado-Rodriguez M et al Nutr Metab Cardiovasc Dis. 2011; 21(4):237–44 doi: 10.1016/j.numecd.2009.10.005 20096543 10.1016/j.numecd.2009.10.005

[42] Pancallo MT, Saulle R, Semyonov L, Amadei P, La Torre G. Effectiveness of a protocol treatment for overweight/obese patients (SIAN—ASL RMA). Clin Ter 2015; 166(5):e306–11. doi: 10.7417/T.2015.1883 26550814 10.7417/T.2015.1883

[43] Stamler J, Berkson DM, Cohen DB, Epstein MB, Frankel JJ, Hall Y et al Prevention of atherosclerotic coronary heart disease by change of diet and mode oflife In Boerhaave Course on Ischaemic Heart Disease, 1969, Leiden. Ed. by Snellen H. A. Springer-Verlag New York, Heidelberg, and Berlin, 1969.

[44] La Torre G, Mannocci A, Saulle R, Sinopoli A, D'Egidio V, Sestili C et al Improving knowledge and behaviors on diet and physical activity in children: results of a pilot randomized field trial. Ann Ig. 2017;29(6):584–594. doi: 10.7416/ai.2017.2187 29048455 10.7416/ai.2017.2187

[45] Mahmood S, Perveen T, Dino A, Ibrahim F, Mehraj J. Effectiveness of school-based intervention programs in reducing prevalence of overweight. Indian J Community Med 2014;39(2):87–93. doi: 10.4103/0970-0218.132724 24963224 10.4103/0970-0218.132724 PMC4067935

[46] Mura G, Rocha NB, Helmich I, Budde H, Machado S, Wegner M et al Physical activity interventions in schools for improving lifestyle in European countries. Clin Pract Epidemiol Ment Health 2015;11(Suppl 1 M5):77–101. 25834629 10.2174/1745017901511010077 PMC4378026

